# Categorial Compositionality: A Category Theory Explanation for the Systematicity of Human Cognition

**DOI:** 10.1371/journal.pcbi.1000858

**Published:** 2010-07-22

**Authors:** Steven Phillips, William H. Wilson

**Affiliations:** 1 Mathematical Neuroinformatics Group, Human Technology Research Institute, National Institute of Advanced Industrial Science and Technology (AIST), Tsukuba, Ibaraki, Japan; 2 School of Computer Science and Engineering, The University of New South Wales, Sydney, New South Wales, Australia; University College London, United Kingdom

## Abstract

Classical and Connectionist theories of cognitive architecture seek to explain systematicity (i.e., the property of human cognition whereby cognitive capacity comes in groups of related behaviours) as a consequence of syntactically and functionally compositional representations, respectively. However, both theories depend on *ad hoc* assumptions to exclude specific instances of these forms of compositionality (e.g. grammars, networks) that do not account for systematicity. By analogy with the Ptolemaic (i.e. geocentric) theory of planetary motion, although either theory can be made to be consistent with the data, both nonetheless fail to fully explain it. Category theory, a branch of mathematics, provides an alternative explanation based on the formal concept of adjunction, which relates a pair of structure-preserving maps, called functors. A functor generalizes the notion of a map between representational states to include a map between state transformations (or processes). In a formal sense, systematicity is a necessary consequence of a higher-order theory of cognitive architecture, in contrast to the first-order theories derived from Classicism or Connectionism. Category theory offers a re-conceptualization for cognitive science, analogous to the one that Copernicus provided for astronomy, where representational states are no longer the center of the cognitive universe—replaced by the relationships between the maps that transform them.

## Author Summary

Our minds are not the sum of some arbitrary collection of mental abilities. Instead, our mental abilities come in groups of related behaviours. This property of human cognition has substantial biological advantage in that the benefits afforded by a cognitive behaviour transfer to a related situation without any of the cost that came with acquiring that behaviour in the first place. The problem of systematicity is to explain why our mental abilities are organized this way. Cognitive scientists, however, have been unable to agree on a satisfactory explanation. Existing theories cannot explain systematicity without some overly strong assumptions. We provide a new explanation based on a mathematical theory of structure called Category Theory. The key difference between our explanation and previous ones is that systematicity emerges as a natural consequence of structural relationships between cognitive processes, rather than relying on the specific details of the cognitive representations on which those processes operate, and without relying on overly strong assumptions.

## Introduction

For more than two decades, since Fodor and Pylyshyn's seminal paper on the foundations of a theory of cognitive architecture (i.e., roughly, the component processes and their modes of composition that together comprise cognitive behaviour) [1], the problem of explaining systematicity has remained unresolved [2] despite numerous Classicist and Connectionist attempts [3]–[7]. In general terms, the problem of systematicity for a theory of cognition is to explain why various cognitive abilities are intrinsically connected in the sense that the capacity to exhibit some abilities is indivisibly linked to the capacity to exhibit some other related abilities. Why, for example, is it the case that if one has the ability to infer that *John* is the lover from *John loves Mary*, then one also has the ability to infer that *Mary* is the lover from *Mary loves John*, where both abilities involve a common relation, *loves*? That is to ask, in general: what is it about our cognitive system that necessitates a particular group-oriented distribution of cognitive capacities, whereby you don't find people with the capacity for some but not all the behaviours pertaining to the same group (excluding, of course, individuals who lack a particular capacity for reasons clearly unrelated to normal development, because of brain damage for example)? Although the debate over what systematicity implies for a theory of cognition has many aspects (see [2]), the generally accepted common ground is that: systematicity is a property of some (though not all) components of human cognition; a complete theory of human cognitive architecture must include an explanation for this property; and no theory of cognition has a satisfactory explanation for it. In the remainder of this section, we outline the systematicity property and the main problem it still poses for existing theories, what is required for a theory to explain it, and how our approach meets those requirements.

The systematicity problem consists of three component problems:

1. *Systematicity of representation*—why is it the case that the capacity to generate some representations (e.g., the representation John loves Mary) is intrinsically linked to the capacity to generate some other representations (e.g., the representation Mary loves John)?
2. *Systematicity of inference*—why is it the case that the capacity to make some inferences (e.g., that John is the lover in the proposition John loves Mary) is intrinsically linked to the capacity to make some other inferences (e.g., that Mary is the lover in the proposition Mary loves John)?
3. *Compositionality of representation*—why is it the case that the capacity for some semantic content (e.g., the thought that John loves Mary, however that thought may be represented) is intrinsically linked to the capacity for some other semantic content (e.g., the thought that Mary loves John, however that thought may also be represented)?

These problems are logically independent—one does not necessarily follow from another [8], and so a theory is required to explain all three, though for some theories an explanation for one property may entail explanations for others.

Classicists and Connectionists employ some form of combinatorial representations to explain systematicity. For Classicists, representations are combined in such a way that the tokening of complex representations entails the tokening of representations of their constituent entities, so that the syntactic relationships between the constituent representations mirror the semantics ones—systematicity is a result of a combinatorial syntax and semantics [1]. For Connectionists, representations of complex entities are constructed more generally so that their tokening does not necessarily imply tokening constituent entity representations [5], [6]. An example of a Classicist's representation of John loves Mary would be loves (John, Mary), and a Connectionist representation would be a tensor product so that the vectors representing John, loves, and Mary do not literally appear anywhere in the tensor representation. We refer to the former as *classical compositionality*, and the latter as *connectionist* (or, *functional) compositionality*.

In general, a Classical or Connectionist architecture can demonstrate systematicity by having the “right” collection of grammatical rules, or functions such that one capacity is indivisibly linked to another. Suppose, for example, a Classical system with the following three rules:

G1:
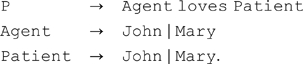



G1 provides the capacities to generate all four representations (i.e., John loves John, John loves Mary, etc.), and these capacities are indivisibly linked, because absence of any one of those rules means the system cannot generate any of those representations. In no case can the system generate one without being able to generate the others. So, this Classical architecture has the systematicity of representation property with respect to this group of four propositions. A tensor product [9], or Gödel numbering [5] scheme is a functionally compositional analogue of this explanation. Systematicity of inference follows from having additional processes that are sensitive to the structure of these representations. For Classical architectures, at least, compositionality of representation also follows, because the semantic content of a complex representation is built up from the semantic contents of the constituents and their syntactic relationships [8]. Aizawa [2], [8] disputes whether a Connectionist architecture can also demonstrate compositionality of representation. Regardless, though, neither Classicism, nor Connectionism can derive theories that provide a full account of systematicity [2].

A demonstration of systematicity is not an explanation for it. In particular, although grammar G1 has the systematicity of representation property, the following grammar:

G2:
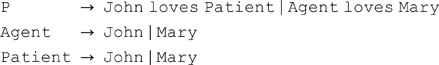
does not. This architecture cannot generate a representation of the proposition Mary loves John even though it can generate representations of both John and Mary as agents and patients, and the John loves Mary proposition. The essential problem for Classical theory—likewise Connectionist theory—is that syntactic compositionality by itself is not sufficient without some additional assumptions for admitting grammars such as G1 that have the systematicity property, while excluding grammars such as G2 that do not. An explanation for systematicity in these cases turns on the nature of those additional, possibly *ad hoc* assumptions.

### An explanatory standard for systematicity

To further clarify what is required of a theory to explain systematicity [1], [3], Aizawa [2] presents an explanatory standard for systematicity and the problem of *ad hoc* assumptions, which we follow, by analogy with the Ptolemean (geocentric) versus Copernican (heliocentric) explanations for the motions of the planets (see [10] for a review). The geocentric explanation for planetary motion places the Earth at the center of the other planets' circular orbits. Although this theory can roughly predict planetary position, it fails to predict periods of apparent retrograde motion for the superior planets (i.e. Mars, Jupiter, etc.) across the night sky without the assumption of *epicycles* (i.e., circular orbits with centers that orbit the Earth). This additional assumption is *ad hoc* in that it is unconnected with the rest of the theory and motivated only by the need to fit the data—the assumption could not be confirmed independently of confirming the theory. The heliocentric explanation, having all planets move around the Sun, eschews this *ad hoc* assumption. Retrograde motion falls out as a natural consequence of the positions of the Earth and other planets relative to the Sun. Tellingly, as more accurate data became available, the geocentric theory had to be further augmented with epicycles on epicycles to account for planetary motion; not so for the heliocentric theory.

The theory of planetary motion, of course, does not end there. The heliocentric theory, with its circular orbits, cannot explain the elliptical motion of the planets without further assumptions, and so was superseded by Newtonian mechanics. Newtonian mechanics cannot explain the precession of planetary orbits, and was in turn superseded by Einstein's theory of relativity. In each case, the superseding theory incorporates all that was explained by the preceding theory. Evaluating competing theories in this manner has an extensive history in science, and so one may expect it to be a reasonable standard for an explanation of systematicity in cognitive science.

Aizawa [2] notes that although philosophers of science may not have a precise definition for the concept of an *ad hoc* assumption, one can nonetheless usefully characterize the idea by analogy with generally accepted examples, such as the assumption of epicycles, which we just mentioned. Another example Aizawa uses is the Creationist versus Darwinian theory of speciation, where the appeal to a supernatural being to explain the existence of different species is an *ad hoc* assumption. The general sense in which a theory fails to provide a satisfactory explanation by its appeal to *ad hoc* assumptions is when those additional, so called auxiliary, assumptions are unconnected to the core assumptions and principles of the theory, motivated only by the need to fit the data, and cannot be confirmed independently of confirming the theory. In this sense, the core theory has no explanatory power for the particular phenomenon of interest. Note that an auxiliary assumption is not necessarily *ad hoc*, nor is it precluded from subsequent inclusion into the set of core assumptions of the modified theory. Orthogonal experiments may provide confirmatory data for an auxiliary assumption, independent of the theory in question. Observations of the Jovian moons would have been the sort of independent confirmatory evidence for epicycles, had such data been available at the time, to justifiably include it as one of the core assumptions. However, the assumption that all heavenly bodies are governed this way ultimately proved untenable. The kind of theory sought here is one where systematicity necessarily follows without requiring such *ad hoc* assumptions. This characterization guides our analysis of the problem posed by the systematicity property, and our explanation for it.

The problem for Classical and Connectionist theories is that they cannot explain systematicity without recourse to their own *ad hoc* assumptions [2]. For Classicism, having a combinatorial syntax and semantics does not differentiate between grammars such as G1 and G2. For Connectionism, a common recourse to learning also does not work, whereby systematicity is acquired by adjusting network parameters (e.g., connection weights) to realize some behaviours—training set—while generalizing to others—test set. Learning also requires *ad hoc* assumptions, because even widely used learning models, such as feedforward [11] and simple recurrent networks [12], fail to achieve systematicity [13]–[17] when construed as a degree of generalization [18], [19]. Hence, neither Classical nor Connectionist proposals satisfy the explanatory standard laid out by Fodor and Pylyshyn [1] and Fodor and McLaughlin [3] (see also [20], Appendix), and further articulated by Aizawa [2]. Ironically, failure to meet this criterion was one of the reasons Classicists rejected Connectionist explanations for systematicity. The import of Aizawa's analysis is that the same shortcoming also befalls Classicism, and so an explanation for systematicity is still needed. In this regard, it would appear that the 90s were also the “lost decade” for cognitive science.

In hindsight, the root of the difficulty that surrounds the systematicity problem has been that cognitive scientists never had a theory of structure to start with (i.e. one that was divorced, or at least separated from specific implementations of structure-sensitive processes). In fact, such a theory has been available for quite some time, but its relevance to one of the foundational problems of cognitive science has not previously been realized. Our category-theory based approach addresses the problem of *ad hoc* assumptions because the concept of an *adjunction*, which is central to our argument, ensures that the construct we seek not only exists, but is unique. That is to say, from this core assumption and category theory principles, the systematicity property necessarily follows for the particular cognitive domains of interest, because in each case the one and only collection of cognitive capacities derived from our theory is the systematic collection, without further restriction by additional (*ad hoc*) assumptions.

## Methods

Category theory is a theory of structure *par excellence* [21]–[23]. It was developed out of a need to formalize commonalities between various mathematical structures [24], and has been used extensively in computer science for the analysis of computation [25]–[28]. Yet, despite computationalism being the catchcry of many psychologists since the cognitive revolution, applications of category theory to cognitive psychology have been almost non-existent (but, see [29], [30] for two examples). Our explanation of systematicity is based on the concept of an *adjunction*, which depends on the concepts of *category*, *morphism*, *product*, *functor*, and *natural transformation*. So, in this section, we provide formal definitions of these concepts. (For further explanation of some category theory concepts in the context of cognition, see [30].)

An adjunction is a formal means for capturing the intuition that a relationship between mathematical objects is “natural”—additional constructs are unnecessary to establish that relationship (see also [23], p2). The mathematical notion of being natural dates back at least to [24], and the technical aspect is given starting where we define *natural transformation*. In the current context of meeting the explanatory standard for systematicity, identifying a suitable adjunction means that no further (*ad hoc*, or arbitrary) assumptions are needed to define the relationship between a particular cognitive architecture and a desired group of cognitive capacities. Such constructs *look* natural (once understood), but it is the mathematical criterion that definitely establishes naturality.

### Category

A *category* 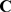 consists of a class of objects 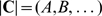; a set 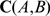 of morphisms (also called arrows, or maps) from 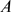 to 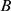 where each morphism 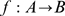 has 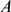 as its domain and 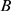 as its codomain, including the *identity* morphism 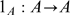 for each object 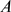; and a composition operation, denoted “
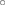
”, of morphisms 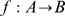 and 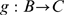, written 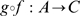 that satisfy the laws of:

- *identity*, where 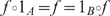, for all 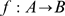; and
- *associativity*, where 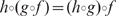, for all 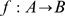, 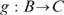 and 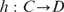.

The most familiar example of a category is 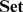, which has sets for objects and functions for morphisms, where the identity morphism 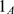 is the identity function and the composition operation is the usual function composition operator “
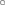
”. Another example, where continuity is important, is the category of metric spaces and continuous functions.

### Morphisms

Certain morphisms have important properties that warrant giving them names. Two such morphisms, which we will refer to later, are called isomorphisms and homomorphisms. A morphism 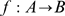 is an *isomorphism* if there exists a morphism 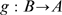, such that 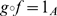 and 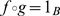. If 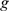 exists, then it is the *inverse* of 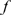, also denoted as 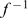.

Homomorphisms pertain to categories whose objects have additional internal structure, such as groups. For example, the category 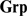 has groups for objects, and the morphisms are group homomorphisms. A group consists of a set 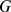 of elements, and an associative binary operation 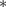, satisfying identity and inverse axioms. That is, 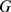 has an identity element 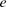, and for each 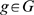, an inverse element 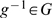, such that 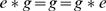 and 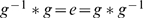. A *group homomorphism* is a morphism 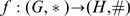, such that 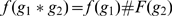, for all 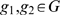. Homomorphisms in other categories (e.g., graph homomorphisms) are defined analogously.

### Product

A *product* of two objects 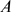 and 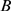 in a category 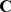 is an object 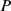 together with two morphisms 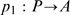 and 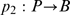, such that for any pair of morphisms 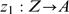 and 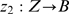, there is a unique morphism 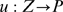, such that the following diagram *commutes*:
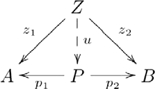
(1)where a broken arrow indicates that there exists exactly one morphism making the diagram commute. To say that a diagram commutes is to mean that the compositions along any two paths with the same start object and the same finish object are the same. So, in this diagram, 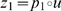 and 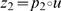, where 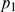 and 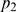 are sometimes called projection morphisms. A product object 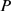 is *unique up to a unique isomorphism*. That is, for any other product object 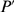 with morphisms 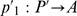 and 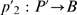 there is one and only one isomorphism between 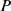 and 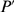 that makes a diagram like this one commute. Hence, 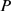 is not unique, only unique with respect to another product object via isomorphism. This characteristic has an important consequence for our explanation of systematicity, which we present in the Results section. An essential characteristic of a product object is that the constituents 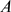 and 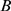 are retrievable via the projection morphisms. 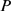 is also written 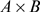, and since 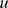 is uniquely determined by 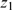 and 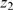, 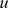 is often written as 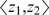, and the diagram used in defining a product then becomes
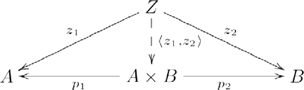
(2)


In 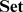, 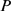 is (up to isomorphism) the Cartesian product (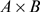, 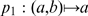, 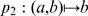), where 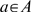, 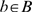, and 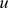 is the product function 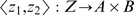, sending 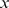 to 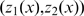, so that 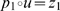 and 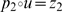. The “maps to” arrow, 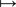, indicates the action of a function on a domain element, so 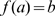 is equivalent to 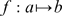. (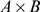 refers both to a general product in any category with products and the more specific Cartesian product in the category 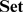.)

The categorical concept of product is a very general notion of combinatoriality. Not surprisingly, then, Classical and Connectionist notions of combinatoriality can be seen as special cases of categorical products. A grammar like G1 (Introduction), for instance, can be used to realize the Cartesian product of the set of agents and the set of patients (i.e. by employing the first production without the loves symbol). A categorical product can also be realized by including suitable rules for inferring the agent and patient from this Cartesian product. (A grammar like G2 cannot realize a Cartesian product, or categorical product; in fact, it realizes a union of two partial products.) Similarly, a Connectionist method such as the outer product of two vector spaces with suitable projections from the outer product space to the original vector spaces also realizes a categorical product. However, an explanation for systematicity requires more than just realization, and as we shall see, additional category theory concepts are needed.

### Functor

A functor 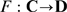 is a structure-preserving map between categories 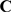 and 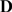 that associates each object 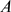 in 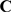 to an object 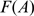 in 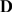; and each morphism 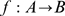 in 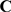 to a morphism 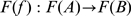 in 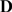, such that 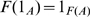 for each object 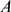 in 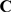; and 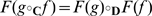 for all morphisms 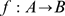 and 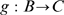 for which compositions 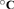 and 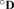 are defined in categories 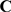 and 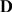, respectively. The following diagram shows the details of a functor:
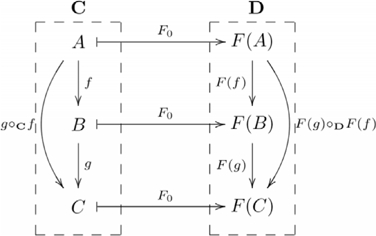
(3)where dashed rectangles encapsulate the categories, and arrows between morphisms are omitted. The object and morphism components of a functor are sometimes explicitly distinguished as 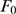 and 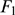, respectively. Otherwise, the functor component is implicitly identified by its argument.

Functor composition and isomorphism are defined analogously to morphisms (above). That is, the composition of functors 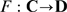 and 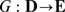 is the functor 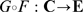, sending all objects 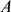 in 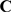 to objects 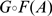 in 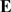; and morphisms 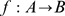 in 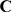 to morphisms 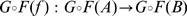, such that identity and composition are respected. That is, 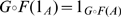; and 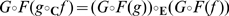. A functor 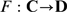 is an *isomorphic functor*, if and only if there exists a functor 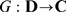 such that 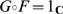 and 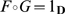, where 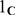 and 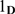 are the identity functors sending objects and morphisms to themselves in the respective categories.

Theories of cognition employ some form of representation. Functors provide a theoretical basis for constructing representations. For example, computational systems often employ lists of items, such as numbers. In category theory, lists can be modeled as monoids from the category 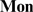 whose objects are monoids, and morphisms are monoid homomorphisms [28]. A *monoid* 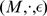 is a set 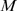, with an associative binary operation , and an identity element 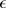, such that 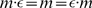 for all 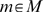. A *list monoid* 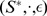 [28] is the set 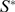 of all ordered lists constructed from set 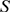 by concatenation operator , where the identity element 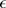 is the empty list (so that, e.g., 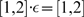). (It is worth noting that strings, e.g., lists of characters, of length 2 over the set 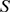 are denoted 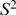, and strings of length 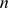 denoted 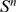. In computer science, 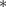 often means “match anything”, hence the notation 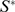 can be read as strings of any length 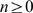.) Lists can be constructed from sets by the functor 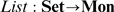, as indicated in the example diagram
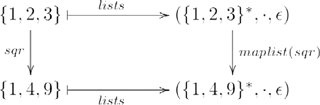
(4)where 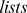 is the object part of 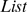 (i.e., 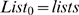) and 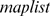 is the morphism part (i.e., 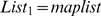), so that, e.g., 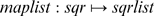 (i.e., morphism 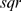 is mapped to monoid homomorphism 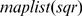, which we will refer to as 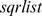). (For simplicity, we have omitted composition with a second morphism in each of the categories and functor mappings, as was shown in Diagram 3.) So, for example, 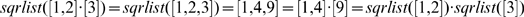. The examples pertaining to lists were adapted from [28] (Chapter 2), where 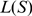 in [28] corresponds to our 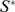. We choose to label the object component of the functor 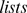 rather than 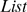 to emphasize the fact that the functor constructs a set of lists of numbers from a set of numbers, not just a single list containing those numbers.

The two different sorts of arrows in Diagrams 3 and 4 highlight the constructive nature of functors. The objects are (co)domains with respect to the morphisms within categories, but are themselves elements of larger objects (in general, the class 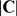) with respect to the morphisms between categories. In programmer parlance, 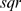 was “lifted” from being a function over numbers to become a function 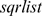 over lists of numbers. In this way, functors provide a means for constructing new representations and processes from existing ones in a structurally consistent manner.

Notice that the definition of functor does not dictate a particular choice for monoid homomorphism as part of the definition of 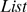. A natural choice is to define 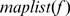 so that functions applied to one-item lists result in one-item lists (i.e., 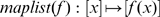). Another choice that turns out to also respect the definition of a functor includes *two* copies of each transformed element (i.e., 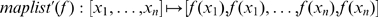). In this case,
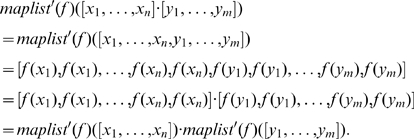
So, 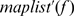 and in particular 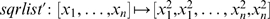 are monoid homomorphisms. In fact, there are many possible monoid homomorphisms that could be chosen to define this functor. Consequently, in the case of an architectural component of a cognitive system, there are many possible ways of constructing structurally consistent representations and processes from existing ones. We need to find a principled way to choose the “right” monoid homomorphism. In the context of explaining systematicity, a similarly principled choice is necessary. To narrow the choice down to a particular monoid homomorphisms, and hence a particular representational scheme, we need two additional category theory concepts: natural transformation and adjunction.

### Natural transformation

A *natural transformation* 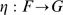 is a structure-preserving morphism from domain functor 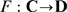 to codomain functor 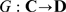 that consists of 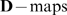 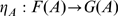 for each object 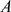 in 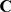, such that 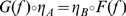, as indicated by the commutative diagram in the category 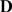


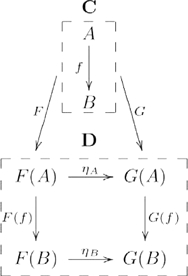
(5)


Again for expository purposes, we include the source category and functor arrows, which are usually left implicit in such diagrams. When a transformation is natural in the technical sense it seems natural in the intuitive sense, for mathematicians. In fact, category theory was founded in an attempt to formalize such intuitions [24]. We will return to this point about naturality, in the Discussion, as it pertains to an explanation of systematicity without reliance on *ad hoc* assumptions.

A natural transformation is a *natural isomorphism*, or *natural equivalence* if and only if each 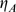 is an isomorphism. That is, for each 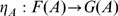 there exists a 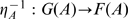 such that 
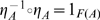
 and 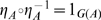. Natural transformations also compose, and the composition of two natural transformations is also a natural transformation. Just as there are identity morphisms mapping objects to themselves, and identity functors mapping categories to themselves, there are also identity natural transformations, 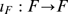, mapping functors to themselves. And, so, the composition of a natural isomorphism (isomorphic natural transformation), 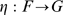, with its inverse, 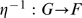, is an identity natural transformation, i.e., 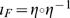.

Functors preserve structure between categories; natural transformations identify the similarities between functors. For our purposes, functors construct new representations and processes from existing ones; natural transformations identify the similarities between constructions. A simple example that is closely related to the 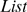 functor example, illustrating this perspective, involves list reversal as indicated by the commutative diagram
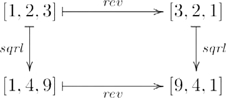
(6)where the domain and codomain objects of each morphism are sets of lists, such as 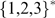; and 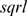 is essentially 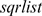 with (co)domain the set 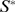 instead of the monoid 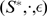. As the diagram illustrates, squaring a reversed list is the same as reversing a squared list. So, there is a non-trivial (i.e. non-identity) relationship between the list monoid construction functor (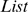) and itself. The functor 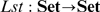 constructing the lists in Diagram 6 is closely related to 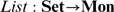 in that the returned object 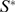 is just the underlying set of the monoid 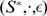, forgetting the binary operation  and the identity element. The underlying set can also be extracted by a functor from the category 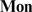, as we will see in the next section. This example shows how two ways of constructing individual lists, via the 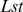 functor, are related by the list reversal natural transformation, 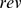.

Although their associated diagrams look similar, there is an important difference between functor and natural transformation pertaining to the equality constraint that defines the relationships between object elements. For a functor, the equality constraint is local to the codomain of the transformation, i.e. the relationships between object elements within the constructed category. And so, the elements of the objects in the new category are only indirectly related to the elements in the corresponding objects of the source category by the categories' common external structure (i.e. inter-object relationships). For a natural transformation, the equality constraint spans the transformation, involving object elements mapped by both domain and codomain functors. And so, the two functors are directly related to each other by the internal structure of their associated objects (i.e. the relationships between object elements within an object). As part of a theory of cognitive architecture, there is a tension between the freedom afforded by functorial construction on the one hand—allowing an architecture to transcend the specific details of the source elements to realize a variety of possible representational schemes for those elements—and the need to pin down such possibilities to specific referents on the other. This tension is resolved with adjunctions.

### Adjunction

An *adjunction* consists of a pair of functors 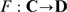, 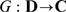 and a natural transformation 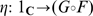, such that for every 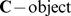 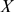 and 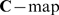 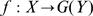 there exists a *unique* 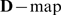 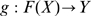, such that 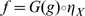, indicated by the following commutative diagram:
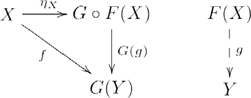
(7)where the functors are implicitly identified by (co)domain categories 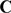 (left subdiagram) and 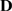 (right subdiagram). The two functors are called an *adjoint pair*, 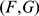, where 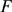 is the *left adjoint* of 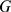, and 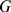 is the *right adjoint* of 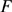; and natural transformation 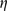 is called the *unit* of the adjunction.

The left and right functors of an adjoint pair are like “inverses” of each other, but unlike an isomorphic functor whose composition with its inverse sends all objects and morphisms to themselves, the returned objects and their elements of a composition of left and right adjoints are related to the argument (source) objects and their elements by a natural transformation. For categories 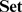 and 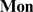, the adjoint pair 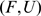, consisting of functor 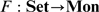 that constructs the *free monoid* 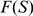 on the set 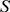, and then “forgetful” functor 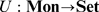 returns the underlying set 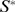 of monoid 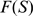, are related by an injection. The injection is called an *insertion of generators*, whose component at 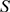, 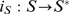, sends each element of 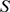 to the corresponding element (one-item list) in 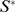. The elements 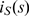 together generate the set 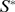 (i.e. 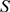 is the alphabet from which the set 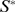 of all “words” is constructed where each 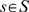 is mapped to 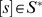). In this context, 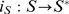 is the unit of this adjoint pair.

The effect of 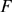 on objects has just been given; the effect on morphisms is as follows: if 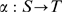 is a function, then 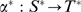 is defined as follows:
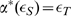


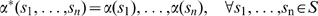
(cf. [25], p.111–112). Note that 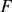 is the functor 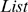 defined in the Functors section.

Monoid 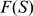 is “free” in the informal sense that there are no missing or extra bits in the construction used to satisfy commutativity. The precise definition of *free* is as follows. Given the forgetful functor 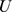, and an object 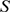 of 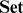, 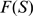 is free on 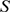 if there is a morphism 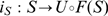 such that for any morphism 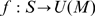, there exists a unique morphism 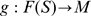 such that 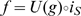, indicated in the following commutative diagram:
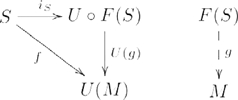
(8)


However, not just any monoid generated from a set is a free monoid. For instance, the monoid 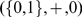 (i.e. addition modulo 2) in the diagram
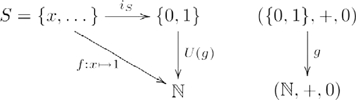
(9)is not the free monoid on any set 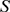, because the only homomorphism, 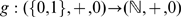, maps 0 and 1 to 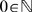, which does not make the diagram commute for 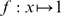. That is, 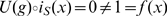. (It is easy to show that the free monoid on the empty set is 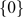. So 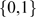 is not the free monoid on the empty set, either.) Other free objects, such as the *free group* on a set are defined analogously (see [21]). A simple example of a free monoid as may be employed by a cognitive system is a primitive form of counting, where 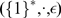 is the free monoid counter, having elements 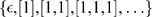, on singleton set 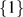. This monoid is isomorphic to addition over the natural numbers, i.e. the monoid 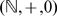.

From free objects we get an alternative (equivalent) definition of adjunction: consider functor 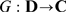 from the original definition. If for every object 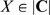, 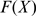 is *free* on 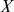 with morphism 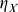, then functor 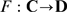, with morphism mappings defined so that 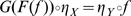, is the left adjoint of 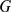, and 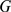 is the right adjoint of 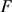 [31].

Yet another (equivalent) definition of adjunction, favoured by category theorists for its conceptual elegance, highlights the symmetry between a pair of adjoint functors: a bijection (one-to-one correspondence) between the set of morphisms from object 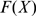 to 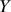 in category 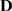 and the set of morphisms from object 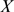 to 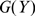 in category 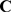. So, identifying the unique morphism in one category means that it is associated with one and only one morphism in the other category.

In the list construction example, the unit of the adjunction is the injection 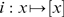 sending each element 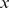 in the set 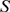 to the one-item list 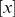 in the set of all lists 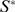 constructed from 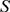, as shown in the following diagram:
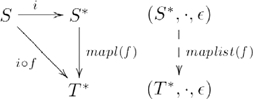
(10)where the left adjoint, 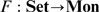, constructs the *free* monoid 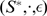 on the set 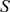; and the right adjoint, 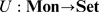, returns the underlying set, 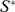, of a list monoid, as mentioned earlier. In this way, given 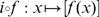, the only homomorphism in the constructed category making the diagram commute is 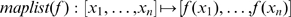. The definition for arrow 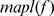 is essentially the same as 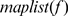, except that its (co)domain is a set, not a monoid. Other monoid homomorphisms that could have been chosen as part of the 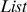 functor definition, such as 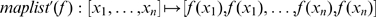, are excluded by 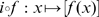 and the commutativity property of the adjunction, because 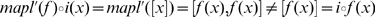.

Since this arrangement works for any morphism in 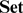, it can also be used to define a particular list length function from a family of analogous “length” functions as indicated in the following commutative diagram:
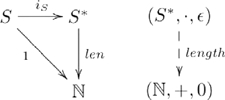
(11)where monoid 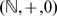 is the set of non-negative integers with addition as the operator and 0 as the identity element; 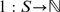 is a constant function sending every element to the number 1; and 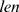/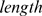 are functions returning the number of items in a list. As in the previous example, the definition of functor affords other choices for “length”, such as 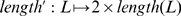, where 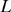 is a list. This arrow is also a monoid homomorphism, since 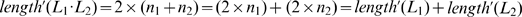, where 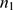 and 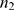 are the lengths of lists 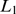 and 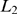, respectively. Again, however, the morphism 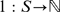 and the commutativity property force the usual choice for length function (i.e. 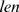), and excludes others such as 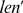, because 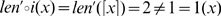.

A general pattern emerges from this use of adjunction. Functor construction may afford multiple choices for particular morphisms (processes) in the constructed category, but a principled choice is obtained through the commutativity property of the adjunction. This arrangement means that we are not committed *a priori* to a particular representational scheme; i.e., we do not have to make an *ad hoc* assumption about what that representational format should be. Given that an architecture has the capacity for an instance of the group of computations under consideration, then necessarily it applies to all other computations in that group. In the case of list length, for example, 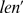 may indeed be the “correct” choice when we require the length of a list of characters in number of bytes for characters that are 2-byte unicodes (i.e. the characters appearing in the extended set that includes other special symbols and language scripts requiring two bytes for unique identification). So, to paraphrase, a computational architecture with the capacity to count the length (in bytes) of some lists of 2-byte unicodes necessarily has the capacity to compute byte lengths for all other unicode lists. In this way, the explanation for the “systematicity of list length” has two parts: existence is afforded by the possible list length functions; and uniqueness is afforded by the commutativity property of the adjunction. Without the adjunction, the choice of construction is by *ad hoc* assumption. Our explanation for the systematicity of human cognition follows this pattern.

## Results

With these formal concepts in hand, we now proceed to our explanation of systematicity. We apply our explanation in two domains: systematicity with respect to relational propositions, and systematicity with respect to relational schemas. Then, we contrast our explanation with the Classical and Connectionist ones.

### Systematicity of relational propositions: (*diagonal*, *product*) adjoint

For expository purposes, we develop our adjoint functors explanation from its components. One may wonder whether a simpler category theory construct would suffice to explain systematicity. For this example domain, the components of this adjoint have some systematicity properties, but in and of themselves do not explain systematicity—just as for Classicism and Connectionism, having a property is not the same as explaining it. This bottom-up approach motivates the more complex category theory construct from which the systematicity properties necessarily follow. Our approach has three steps. *First*, we show a categorical product that has the systematicity of representation and systematicity of inference properties. However, a product of two objects may afford many isomorphic product objects that do not also have the compositionality of representation property. *Second*, we show that the product functor provides the principled means for constructing only those products that also have the compositionality of representation property. There may, however, be several products that have the compositionality property, but which differ in semantic content by having different orders between identical sets of constituents. So, a principled choice is needed to determine *the* product. So, *third*, we show that the diagonal functor, which is left adjoint to the product functor, provides that principled choice by the commutativity property of the (diagonal, product) adjoint functor pair. For concreteness, we refer to the category 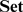, but our explanation does not depend on this category.

(If we require an explanation of systematicity with respect to ternary relational propositions, then a ternary product 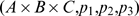 is employed. The explanation for systematicity extends analogously, where the diagonal and product functors involve object triples. We may also need to explicitly represent a symbol for a relation, such as Loves. In this case, an object representing the relation symbol is paired with the product object representing the related entities. We address this situation in the next section. For present purposes, we omit relation symbols, since the relation is constant across the instances considered here and nothing essentially changes by its omission.

First, suppose objects 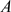 (say, agents) and 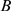 (patients) are sets containing representations of John and Mary, denoted as 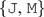. Although 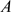 and 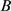 are the same set of members, we maintain distinct names to keep track of the distinction between member pairs. (The assignment of elements to objects is itself an assumption, but not an *ad hoc* one for our theory, as explained in the next section and in the Discussion.) A categorical product of these two sets is the Cartesian product of 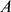 and 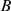, which is the set of all pairwise combinations of elements from 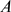 and 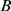, together with projections 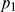 and 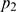 for retrieving the first and second constituents in each case. That is, 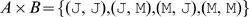, 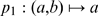, and 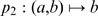. By definition, the Cartesian product 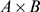 generates all pairwise combinations of elements from 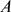 and 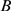, therefore this Cartesian product has the systematicity of representation property. Moreover, by definition, the categorical product 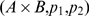 affords the retrieval of each constituent from each representation (otherwise it is not a product), therefore the categorical product also has the systematicity of inference property. In this case, 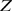 from the categorical product definition takes the role of input, so in terms of Diagram 2 inferring John as the lover from John loves Mary is just 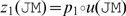, where JM is the input and 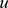 is the input-to-product object map, whose unique existence is guaranteed by definition.

The Cartesian product, however, is not the only product object that satisfies the definition of a categorical product of 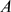 and 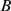. An alternative product has 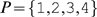 as the product object, and 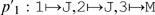 and 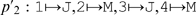 as the projections. Indeed, for this example, any four-item set together with the appropriate projections for retrieving the constituents would suffice. However, these alternatives do not have the compositionality of representation property: the semantic contents of these representations, whatever they may be, are not systematically related to each other, or the semantic content of John, or Mary. Hence, categorical products, in themselves, do not necessarily provide an explanation of systematicity.

Second, for any category 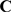 that has products (i.e. every pair of objects in 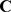 has a product), one can define a product functor 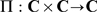 (or, 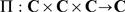, in the ternary case), that is from the Cartesian product of categories, 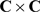, itself a category, to 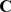, where 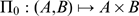, 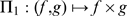, as indicated by the following diagram:
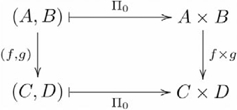
(12)recalling that our functor diagrams explicitly identify the object component, 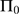, but not the morphism component, 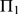, of the functor. In this case, the semantic contents of these elements are systematically related to each other and their constituents John and Mary. This categorical construction is an instance of Classical compositionality, whereby the constituents 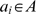, 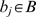 are tokened wherever the compositions 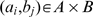 are tokened. As such, it has the compositionality of representation property.

Although the product functor has the compositionality of representation property, it introduces a different problem: 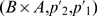, where 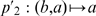 and 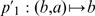 is also a valid product, but the semantic content of 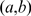 is not the same as 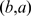. That is because they have different order relationships between their constituents even though the corresponding constituents are identical. Thus, a principled choice is required to determine whether, for example, John loves Mary should map to (John, Mary), or (Mary, John). Otherwise, one can define an architecture that does not have the systematicity of inference property by employing both products to correctly infer Johnas the lover in John loves Mary via 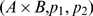, yet incorrectly infer John as the lover in Mary loves John via 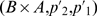, where position within the product triple identifies the relevant projection. The assumption that architectures employ only the first product is *ad hoc* just like the assumption that Classical architectures employ grammars such as G1, but not G2. So, a principled choice is needed to determine *the* product.

Third—final step, this problem brings us to the second aspect of our explanation foreshadowed in the Introduction (i.e. uniqueness). Again, as we saw with lists, a particular construction is specified through the left adjoint functor. The left adjoint to the product functor is the *diagonal* functor 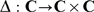 (or, 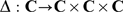, in the ternary case), where 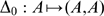, 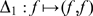 as indicated by the following diagram:
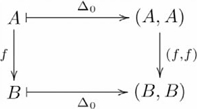
(13)


The (diagonal, product) adjoint pair is indicated by the following commutative diagram:
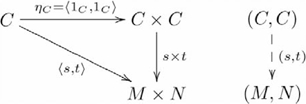
(14)(see [28] Example 2.4.6). In this manner, the John loves Mary family of cognitive capacities is specified by the commutative diagram
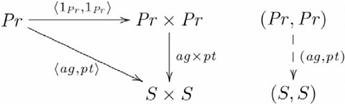
(15)where 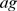 and 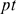 are the *agent* and *patient* maps from the set of proposition inputs 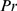 into the set 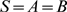 containing all the possible constituent representations. Here, we explicitly consider the case of equality, so that 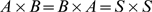. When 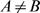, 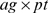 and 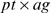 have different codomains, since 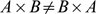, so the conflict between these products does not come into play, therefore the adjunction is not required and the product functor is sufficient. With the understanding that sets 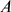 and 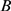 are equal, we maintain the notational distinction for clarity in the subsequent text. Given 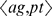 as the morphism used by the architecture to map proposition inputs to their corresponding internal representations, then the definition of an adjunction guarantees that 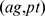 is unique with respect to making Diagram 15 commute via 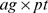. That is, 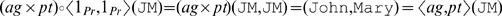, where 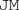 is the input for proposition John loves Mary. The alternative construction 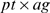 is excluded because 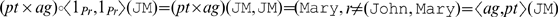. Having excluded 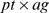 by the commutativity property of the adjunction, the only two remaining ways to map the other inputs (i.e. 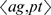 and 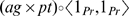) are equal. So, given that the architecture can represent John loves Mary as 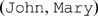 via 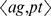 and infer John as the lover via 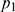 from the product 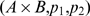, then necessarily it can represent Mary loves John and infer Mary as the lover using the same morphisms. That is, 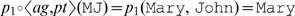, or 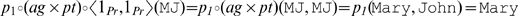.

This explanation works regardless of whether proposition John loves Mary is represented as (John, Mary) via 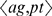, or (Mary, John) via 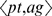. In the latter case, the adjunction picks out just the construction 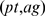, and hence 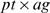, because it is the one and only one that makes the following diagram commute:
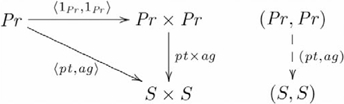
(16)


That is, 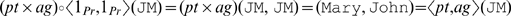,but 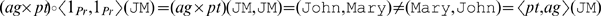. Given that the architecture can represent John loves Mary as 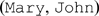 via 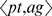 and infer John as the lover via 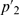 from the product 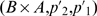, then necessarily it can do so for Mary loves John using the same morphisms. That is, 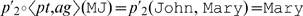, or 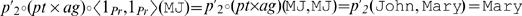.

### Explicit (multiple) relational propositions

If we need to explicitly represent a symbol for a relation, such as Loves, the product object is paired with an object, say 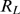, representing the context in which the entities are related. The object representing the relation in this case is 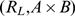. This situation may arise where we need an explanation for systematicity that involves multiple similar relations, e.g., *loves*, *likes*, *dislikes*, and *hates*, where the capacity for instances of each of these relationships is co-extensive. That is, if one can represent John loves Mary *and* John likes Mary, then one can also represent the other six combinations, such as Mary loves John and Mary likes John. If one can represent John loves Mary, but not John likes Mary, then one can represent Mary loves John, but not Mary likes John. In this case, there is a category 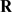 of relation symbols whose objects, 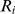, are symbols referring to each relation (e.g., *loves*, *likes*, etc.), and whose morphisms, 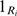, are just the identity morphisms for each object. (Such a category is called a *discrete* category.) Each relation, in this case, is a pair 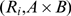. Hence, the capacity to represent instances of the *loves* and *likes* relations extends to the other instances for both relations.

For these situations, the diagonal and product functors have extensions. The extension to the diagonal functor is: 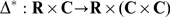, such that 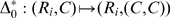 and 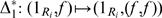. The product functor is: 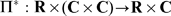, such that 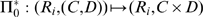 and 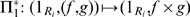. The adjunction, which is an extension of the one shown in Diagram 15, is shown in the following commutative diagram:
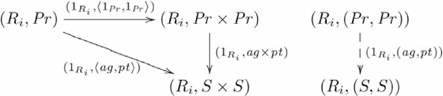
(17)


In this situation, 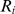 provides the explicit context in which entities are related.

Under the assumption that these relation symbols belong to a different category, then cases such as loves loves loves cannot be generated. Note that supposing different objects for these entities is not an *ad hoc* assumption for our theory. 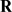 does not contain members such as John or Mary, and likewise 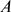 (or, 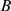) does not contain relation symbols, because they refer to different types of entities with respect to the theory—Loves refers to a relation, which is at the level of objects in our theory, whereas John and Mary refer to entities in a relationship, which are members of objects.

### Summary

In summary, products may have the systematicity of representation and inference properties (see also Discussion), but may not have the compositionality of representation property. Product functors construct products that have the compositionality property, but there may be more than one product with this property. The possible presence of multiple products requires a principled choice for fixing *the* product. That choice is provided by the (diagonal, product) adjoint functor pair. Importantly, the unit of the adjunction, 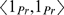, is not a free parameter of the explanation, it defines the specific adjunction in part; and there is no choice in representational format (i.e. left-right, or right-left constituent order)—the given capacity to represent a proposition fixes the same order for all the other propositions. The same situation also applies for the explicit (multiple) relational propositions domain. Hence, systematicity is a necessary consequence of this (extended) adjoint pair without recourse to *ad hoc* assumptions, and so meets the explanatory standard set by Aizawa [2], and Fodor and Pylyshyn [1], for this domain.

### Systematicity of relational schemas: (*free*, *forgetful*) adjoint

Another domain in which humans exhibit systematicity is relational schema induction. This domain is more complex than the previous one in that the intrinsic connection is between relations, rather than within one. In the relational schema induction paradigm [32], participants are required to do cue-response prediction over a set of stimuli, such as letters and shapes, whose relationships conform to a group-like structure. For example, participants are shown (trigram, shape) pairs generated from a set of four trigrams (e.g., NEJ, POB, KEF, BEJ) and two shapes (e.g., square, circle), and are required to predict the response trigram, also from the same trigram set. Suppose, for example, a participant is presented with NEJ and square. After making a prediction, the correct response trigram is presented. This procedure is repeated with a new cue-response trial. The first two responses are not predictable prior to the feedback provided by the correct trigram. Hence, the first two trials are regarded as “information” trials. Each block of eight trials (i.e. all possible trigram-shape combinations) is repeatedly presented until a certain criterion level of correct performance is reached (e.g., correct responses to all eight trials in a block). Each set of eight cue-response pairs (i.e., four trigram times two shapes) constitutes a task instance. Once participants reach criterion a new task instance of eight cue-response pairs was randomly generated from a larger pool of possible trigrams and shapes (task instance examples are shown in Tables 1 and 2). The crucial data for this paradigm are the performances on subsequent task instances. When subsequent task instances conformed to the same structure, albeit with different stimuli, mean response error over the 48 participants was at or near optimal level: 2.00 errors per eight trials for the sequence of task instances conforming to the Klein group, and 2.67 for task instances conforming to the cyclic-4 group—two information trials are needed to determine the assignment of novel stimuli to structural elements [32]. The results provide another example of systematicity of human cognition: given that a person can correctly do one task instance and the information trials from the new task instance, then necessarily they can predict trials of all others, with the usual provision for a distinction between competence and performance.

**Table 1:** First task instance.

*acts-on*	NEJ	POB	KEF	BEJ
square	POB	NEJ	BEJ	KEF
circle	BEJ	KEF	POB	NEJ

**Table 2:** Second task instance.

*acts-on*	GUD	QAD	JOQ	REZ
cross	QAD	GUD	REZ	JOQ
triangle	REZ	JOQ	QAD	GUD

This task is modelled as the category of *sets with actions*, 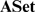 (cf. [25], 6.3.1, and [33] Definition 5.2), that has objects 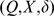 for task instances, where 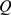 is a set of states indicated by trigrams, 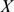 is a set of “actions” indicated by shapes, and 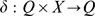 specifies the action of a shape on a trigram resulting in a trigram. The morphisms 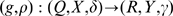 in this category consist of pairs of maps 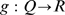 and 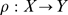, such that the following diagram commutes:
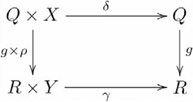
(18)where the identity morphism 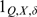 is the pair of identity maps 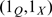, and compositions are defined component-wise. In our example, the set 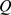 consists of four elements representing the four trigrams, and the set 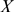 consists of two elements representing the two shapes.

For the purpose of finding a suitable adjoint, we need to see how 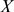 is naturally embedded in a monoid. Recall that a monoid 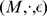 consists of a set 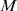 and a binary associative operator  that satisfies closure: i.e., for all 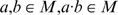, whenever 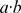 is defined, and there is an identity element 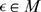, such that 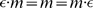. In terms of our ASets (i.e. objects in 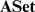), the monoid identity corresponds to a “shape” whose action is to do nothing at all to the trigrams on which it acts: it leaves them unchanged. (However, this shape was not included in the experiments [32].)

The adjoint functor pair used for this domain consists of the *forgetful* functor 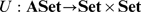, which returns the underlying sets, i.e. 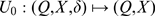 and 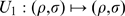, and its left adjoint, the *free* functor 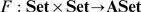, which constructs ASets. The (free, forgetful) adjoint is shown in the following commutative diagram:
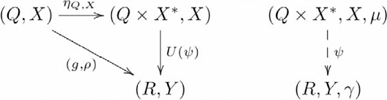
(19)where 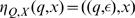 and, for the instance of interest to us, 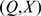 and 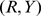 are the (trigram, shape) pairs of sets for the first and second tasks (respectively), as defined for example in Tables 1 and 2 so that 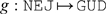, 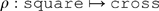, etc. Full details and a proof that 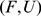 is an adjoint functor pair are provided in Text S1.

Our explanation for systematicity in this domain follows the now familiar pattern, where monoids model the relationships between actions in each task instance. (Though our argument employs monoids, nothing essential changes if instead we use semigroups, or groups, where for example each task instance is extended with two additional shapes, one explicitly corresponding to the identity element, and the other to the remaining element in the Klein, or cyclic-4 group. For these cases, the proofs of adjointness can be extended to involve free semigroups and free groups, respectively.) Given an ASet modelling the first task instance and an ASet modelling the second task instance, there is more than one homomorphism from the first to the second, only some of which afford the correct responses to the stimuli in the second task instance. For example, one homomorphism has the following trigram and shape mappings: 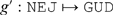, 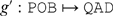, 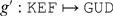, 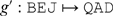, 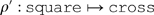, and 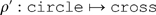. Basically, the <1?show=[to]?>first table collapses to a table with one row and two columns. It is straight forward to check that it is indeed a homomorphism, for example, 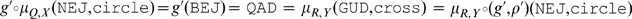. However, this homomorphism does not yield the correct responses to some of the stimuli in the second task instance. For example, all predictions to trigrams REZ and JOQ are no longer possible. Thus, a principled choice is required to select only those homomorphisms that indeed result in models for the second task instance. That choice is determined by 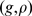 and the commutative property of the adjunction. That is, having obtained the first task instance, and given the two information trials of the second task instance that identify the correspondences between task stimuli, then there is one and only one homomorphism making the diagram commute, so that correct responses are obtained from the remaining trials of the second task instance. And so, systematicity is a necessary consequence of this adjunction.

### Explanatory levels: *n*-category theory

Some readers may be interested in developing alternatives, or extensions to existing theories to address the systematicity problem in light of our explanation, so it is worth formally characterizing how our approach differs from previous ones. The difference between our category theory explanation and Classical/Connectionist approaches to systematicity may be characterized as higher-order versus first-order theories. Category theory also provides a formal basis for this distinction in terms of more general *n*-category theory (see, e.g., [34]). Though the concerns of *n*-category theorists go way beyond what we need here, some elementary aspects of the theory are used to formalize the difference between why our adjoint functors explanation addresses the systematicity problem and why the Classical or Connectionist approach does not.

Notice that the definitions of functor and natural transformation are very similar to the definition of a morphism. In fact, functors and natural transformations are morphisms at different levels of analysis: a natural transformation is a morphism one level above functors as we shall see. For *n*-category theory, a category such as 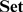 is a 1-category, with 0-objects (i.e. sets) for objects and 1-morphisms (i.e. functions) for arrows. A functor is a morphism between categories. The category of categories, 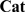, has categories for objects and functors for arrows. Thus, a functor is a 2-morphism between 1-objects (i.e. 1-categories) in a 2-category. A natural transformation is a morphism between functors. The functor category, 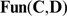 of functors from 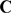 to 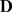, has functors for objects and natural transformations for arrows. Thus, a natural transformation is a 3-morphism between 2-objects (i.e. functors) in a 3-category. (A 0-category is just a *discrete* category, where the only arrows are identities, which are 0-morphisms.) In this way, the order 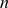 of the category provides a formal notion of explanatory level.

Classical or Connectionist compositionality is essentially a lower-levels attempt to account for systematicity. For the examples we used, that level is perhaps best described in terms of a 1-category. Indeed, a context-free grammar defined by a graph is modelled as the *free category* on that graph containing sets of terminal and non-terminal symbols for objects and productions for morphisms [31]. By contrast, our category theory explanation involves higher levels of analysis, specifically functors and natural transformations, which live in 2-categories and 3-categories, respectively. Of course, one can also develop higher-order grammars that take as input or return as output other grammars. Similarly, one can develop higher-order networks that take as input or return as output other networks (e.g., networks whose connectivity is dynamic, such as cascade correlation [35]). However, the problem is that neither Classical nor Connectionist compositionality delineates those (higher-order) grammars or networks that have the systematicity property from those that do not. Likewise for our category theory explanation, not just any functor, nor just any natural transformation accounts for systematicity. If the explanation was left at either of these levels, then our approach would also succumb to the same problem that befalls Classicism and Connectionism—i.e. the problem of having to stipulate, *ad hoc*, just which functors or natural transformations account for the systematicity property. Rather, it is a natural transformation between an identity functor and a composition of two other functors (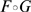) that defines the adjunction that accounts for systematicity relative to the particular domain of interest. In this formal sense, a crucial difference is that there is also a between-levels aspect to our explanation.

## Discussion

Our adjoints explanation of systematicity has essentially two parts: (1) existence, showing how a particular connection between cognitive capacities is possible from a functorial specification of the architecture; and (2) uniqueness, explaining why that particular connection is necessary because it is the one and only one that satisfies the commutativity property of the adjunction. In contrast, the Classical and Connectionist explanations only provide an account of existence, but not uniqueness. That is, some grammars/networks afford the required intrinsic links between capacities and some do not, just like some functorial constructions do and some do not; but, for Classicism or Connectionism, there is no further explanation determining only those grammars or networks yielding systematicity (other than by *ad hoc* assumption), whereas for the category theory explanation the adjunction specifies only the systematic functors. So, our explanation meets the explanatory standard laid out by Aizawa.

To be regarded as a theoretical explanation for systematicity, such an explanation should be potentially falsifiable. Our explanation could be challenged by an alternative theory that accounts for systematicity (without *ad hoc* assumptions) in a way that does not require, or implement an adjunction. This possibility would not falsify our explanation as such, but may provide an alternative theory that is preferred on other grounds. Alternatively, there may exist a domain in which humans exhibit systematicity but for which there does not exist a relevant adjunction. Hence, the category theory approach we have put forward is in principle falsifiable.

The unit of an adjunction is a natural transformation between functors. The sense in which a transformation is natural is that the transformation does not depend on a particular “basis”. A mathematician's example is to contrast the dual of a vector space with the, natural, double dual (dual of the dual) of a vector space—the former depends on a specific set of basis vectors chosen *ad hoc*, the latter does not. The analogue, here, is that our explanation of systematicity is natural in that it does not depend on a particular representational scheme (i.e., constituent order for relational propositions). Hence, the explanation does not depend on *ad hoc* assumptions about internal representations. Contrast this explanation with the Classical one, which must assume a particular grammatical form (e.g., G1 over G2) to fit the data.

In addition to explaining systematicity, our category theory approach has further implications. According to our explanation, systematicity with respect to binary relational propositions requires a category with products. A category theory account has also been provided for the strikingly similar profiles of development for a suite of reasoning abilities that included *Transitive Inference* and *Class Inclusion*, among others [30]—all abilities are acquired around the age of five years. The difference between the difficulties of younger children and the successes of older children (relative to age five) across all these reasoning tasks was explained as their capacity to compute (co)products. (A *coproduct* is related to a product by arrow reversal—see, e.g., [28] for a formal definition.) Therefore, our explanation implies that systematicity is not a property of younger children's cognition. Some support for this implication is found on memory tasks that require binding the background context of memorized items [36], though further work is needed to test this implication directly.

Our explanation for systematicity in regard to binary relational propositions does not depend on 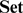, it only requires a category with products. For example, the categories 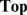 of topological spaces and continuous mappings, and 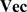 of vector spaces and linear mappings [21] could also be used. These possibilities imply that an explanation of systematicity does not depend on a particular (discrete symbolic, or continuous subsymbolic) representational format. Thus, a further benefit is that our approach opens the way for integration of other (sub/symbolic) levels of analysis.

Though some effort is needed to provide a category theory explanation for systematicity, even for a relatively simple domain such as relational propositions, the potential payoff is that our explanation generalizes to other domains where an appropriate adjunction is identified. This sort of tradeoff has been noted elsewhere in the context of a category theory treatment of automata [25]. We sketch one possibility in the domain of context-free grammars. Languages conforming to context-free grammars can be modelled as the *free category* on the directed graph that defines the grammar, whose vertices are sets of terminal and non-terminal symbols, and edges are transitions [31]. The left adjoint is the functor 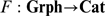 from the category of directed graphs and graph homomorphisms to the category of categories and functors (category homomorphisms). The right adjoint is the forgetful functor 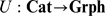, which returns the underlying graph (i.e. the arrows, forgetting their compositions). The explanation here is analogous to our explanation for relational schemas. The problem Aizawa raised with respect to Classicism is avoided here because systematicity is not derived from individual grammars, but homomorphic relationships between grammars.

Having provided an explanation of systematicity in terms of the rather abstract category theory concept of adjoint functors, one may wonder what this explanation means for a more typical conception of cognitive architecture in terms of internal representations and processes, and their realization in the brain. Human cognition is remarkable in that it affords the ability to think about things that have no sensory access (e.g., “a dog that is one lightyear long …”); yet reason about such entities as if they were grounded in our everyday experience (“… is smaller than a dog that is two lightyears long”). However, these two aspects must be reconciled: unbridled abstraction means that one can no longer determine what a particular internal representation is supposed to refer to; yet blinkering the system with over-narrowly defined representations curtails one's ability to *think outside the box*. These aspects appear in the form of functors and natural transformations in category theory. The adjunction is the category theory way of bringing them into precise “synchrony”, or co-ordination, so that we may think abstractly about very specific things.

The realization of computational processes in the brain is classically conceived as a *physical instantiation mapping* from computational states to brain states, where the syntactic relationships between computational states correspond to physical relationships between brain states via such maps (see [1], p13). Category theory affords a similar, but more general and formal treatment in terms of functors. Diagrams of categories are formally defined as functors that map graphs (i.e. the shape of the diagram) to categories (see, e.g., [37]). Analogously, a categorial cognitive system would involve a functor from a categorial computational model to a brain system.

Up to this point, we have not considered the relatively new Bayesian approach to cognitive modelling (see, e.g., [38], [39] for summaries) because, to our knowledge, a Bayesian explanation for systematicity has not yet been articulated. Nonetheless, the hierarchical Bayesian approach offers a significant advance with the ability to learn a diverse range of structures, such as lists, trees, and other (acyclic or cyclic) graphs, from data [40]. An important aspect of this approach is that structural form (or the type of structure) is encoded as prior beliefs by hyperparameters in the higher layers, and instances of those structures are encoded as parameters in the lower layers in so far as they conform to the constraints imposed by the data (environment). In this way, the architecture is not required to presume one particular structure to induce a group of behaviours from data. The hierarchical Bayesian approach affords the sort of higher-order theory that our analysis in the previous section implies. However, the question for the Bayesians is essentially the same as for the Classicists and Connectionists: that is, to articulate the Bayesian architectural principles from which systematicity necessarily follows. As the approach currently stands, systematicity depends on a number of factors including the available data, network connectivity, and optimization parameters. A Bayesian network with independently modifiable parameters for representing the distributions of constituents in each argument position of a relation may not have the systematicity property in the absence of data with, say, Mary in the patient position (so called *strong systematicity* [18]), simply because there may be no (prior) information available to determine the value of the associated parameters. Hyperparameters may enable a dependency between lower level parameters so that the acquisition of one entails the acquisition of another. Still, systematicity may not necessarily follow from hyperparameters alone: for example, one can envisage a network where partial hyperparametrization links some but not all behaviours within the group, analogous to the problem that was raised with respect to classical compositionality.

All theories make certain assumptions. The question is whether those assumptions are extrinsic to the theory and carry the essential explanatory burden (i.e. they are *ad hoc*). In our case, one may question whether supposing that an object contains representations of John and Mary is not itself an *ad hoc* assumption, for the Cartesian product does not necessarily represent all possible combinations of mental representations [41] (e.g., 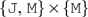 generates representations corresponding to John loves Mary and Mary loves Mary, but not John loves John). Our explanation for systematicity of binary relational propositions is a consequence of the (diagonal, product) adjoint (Diagram 15), not a specific categorical product. Though the categorical product is a component of the explanation, the particular product is derived from the adjunction, not chosen independently of it. Where the constituent entities are of the same sort, and so belong to the same object (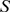) in our theory, the diagonal functor generates the object pair 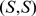, and the product functor takes 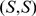 and generates the product object 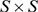, hence cases like 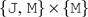 cannot occur in this formulation. The assumption that relation symbols belong to a different category than the related arguments precludes the generation of intrinsically unconnected cases, such as loves loves loves. Typing, in this sense, shares some of the explanatory burden, but types are not extrinsic to our theory. An element cannot exist without belonging to an object (its type) in a category, by definition. Hence, types are intrinsic to the theory. Moreover, the explanatory burden is also born by the adjunction in our example domains. Even with typing, there must still be a principled choice for the order of those constituents, when they involve the same objects, which is provided by the adjunction. And, given that adjunctions are central to category theory, neither the assumption of types, nor our use of adjunction can be regarded as *ad hoc* for the purpose of explaining systematicity in these domains. Classicism also makes a distinction between atomic and molecular representations, as a core assumption [1]. However, even under core assumptions that are equivalent to ours—John and Mary belong to the same word classes, which differ from loves—systematicity does not necessarily follow, as exemplified by grammar G2. Hence, the critical difference between our explanation of systematicity and the Classical approach is the adjunction.

This assumption of typing, though, is acute for quasi-systematic domains, where cognitive capacity may extend to some but not all possible constituent combinations, which appear to be particularly prevalent in language (see [41]). For these cases, we would also need category theory-derived principled restrictions to products. *Equalizers* and *pullbacks* (see [30] for an application to cognitive development) are two ways to restrict (product) objects, in the same arrow-theoretic style. Products, pullbacks and equalizers are all instances of the general, formal concept of a *limit* in category theory. The existence of adjoint functors is closely linked to the existence of limits in the respective categories (cf. *adjoint functor theorems* [21], p210–214), which suggests that an appropriate adjunction can also be found for domains that require an explanation for quasi-systematicity.

Needless to say, our category theory explanation is not the final word on a theory of cognitive architecture. For our approach (and Classicism), where the assignment of elements to objects (and, words to word classes) is asserted, there is also the broader question of why they get assigned in a particular way. This question pertains to the acquisition of representations, whereas the systematicity problem pertains to their intrinsic connections. Incorporating category theory into the Bayesian approach may provide a more integrative theory in this regard. A connection between category theory and probability has been known for some time (see [42]), and category theory concepts have been incorporated into the development of probabilistic functional programming [43]. A potentially fruitful line of future research, then, may be to identify a suitable adjunction with respect to, say, a category of Bayesian models, if such a category exists.

From a category theory perspective, we now see why cognitive science lacked a satisfactory explanation for systematicity—cognitive scientists were working with lower-order theories in attempting to explain an essentially higher-order property. Category theory offers a re-conceptualization for cognitive science, analogous to the one that Copernicus provided for astronomy, where representational states are no longer the center of the cognitive universe—replaced by the relationships between the maps that transform them.

## Supporting Information

Text S1 Proof that the free and forgetful functors for the category ASet form an adjoint functor pair.
(0.10 MB PDF)
