# Supplementary material for: Categorial Compositionality: A Category Theory Explanation for the Systematicity of Human Cognition
Source: PLoS Comput Biol. 2010 Jul 22;6(7):e1000858. doi: 10.1371/journal.pcbi.1000858 (PMC2908697; doi:10.1371/journal.pcbi.1000858)
Supplement: Text S1 — Proof that the free and forgetful functors for the category ASet form an adjoint functor pair. (0.10 MB PDF) [file pcbi.1000858.s001.pdf]

Text S1

Adjunction (proof)

We provide a proof of adjointness adapted from [1] to the more general case where actions also vary. Here, we write $(X^*, \cdot, \epsilon)$ for the free monoid on the set X with binary associative operator $\cdot$ and identity ϵ.

Definition (**ASet**). The category **ASet** (sets with actions) has objects (Q, X, δ) that consist of a set Q, and a set X whose members "act on" members of Q, and a map $\delta : Q \times X \to Q$, which specifies these actions. Thus, if $q \in Q$ and $x \in X$, then $\delta(q, x) \in Q$ is the result of x acting on q. The morphisms of **ASet** are the functions $(g, \rho) : (Q, X, \delta) \to (R, Y, \gamma)$, that is, pairs of maps $g : Q \to R$ and $\rho : X \to Y$, such that the following diagram commutes:

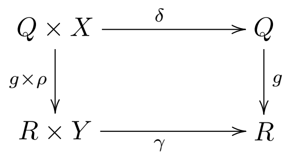

(1)

where the identity morphism $1_{Q,X,\delta}$ is the pair of identity maps $(1_Q, 1_X)$, and compositions are defined component-wise. That is, the composition of $(g, \rho) : (Q, X, \delta) \to (R, Y, \gamma)$ and $(h, \sigma) : (R, Y, \gamma) \to (S, Z, \xi)$ is $(h, \sigma) \circ (g, \rho) : (Q, X, \delta) \to (S, Z, \xi)$, and it is indeed an **ASet** morphism, that is, the following diagram commutes:

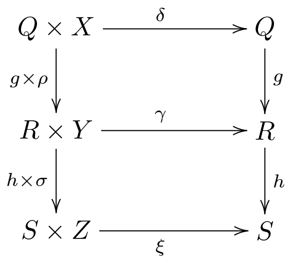

(2)

It is straightforward to prove that **ASet** is a category, by showing the morphisms satisfy the laws of identity and associativity.

Definition (run map). The *run map* of an object (Q, X, δ) is the unique map $\delta^* : Q \times X^* \to Q$,

defined inductively by:

$$\delta^*(q,\epsilon) = q, \quad \forall q \in Q \tag{3}$$

$$\delta^*(q, w\cdot[x]) = \delta(\delta^*(q,w),x), \quad \forall q \in Q, w \in X^*, x \in X. \tag{4}$$

If we regard X as a subset of X^*, i.e. as the part of X^* consisting of "lists" of length 1, then $\delta^*(q,[x]) = \delta^*(q,\epsilon\cdot[x]) = \delta(\delta^*(q,\epsilon),x) = \delta(q,x)$, so δ^* does indeed agree with δ on $Q\times X \subset Q\times X^*$.

It is immediate that if (Q,X,δ) is an ASet, then so is $(Q\times X^*, X, \mu_{Q,X})$, where $\mu_{Q,X} : (Q\times X^*)\times X \to Q\times X^*$, such that $\mu_{Q,X} : ((q,w),x) \mapsto (q, w\cdot[x])$.

Proposition. If (Q,X,δ) is an ASet, then the following diagram commutes:

$$\begin{array}{ccc} (Q\times X^*)\times X & \xrightarrow{\mu_{Q,X}} & Q\times X^* \\ {\scriptstyle \delta^*\times 1_X}\downarrow & & \downarrow{\scriptstyle \delta^*} \\ Q\times X & \xrightarrow[\delta]{} & Q \end{array} \tag{5}$$

That is, $(\delta^*, 1_x)$ is a morphism of ASets.

Proof. For all $q\in Q$, $w \in X^*$, $x\in X$,

$$\begin{aligned} \delta^*\circ\mu_{Q,X}((q,w),x) &= \delta^*(q,w\cdot[x]) && \text{(definition of } \mu_{Q,X}) \\ &= \delta(\delta^*(q,w),x) && \text{(Equation 4)} \\ &= \delta\circ(\delta^*\times 1_X)((q,w),x) && \square \end{aligned}$$

Recall the forgetful functor $U : \mathbf{ASet} \to \mathbf{Set}\times\mathbf{Set}$, such that $U : (T,Z,\zeta) \mapsto (T,Z)$.

Theorem. Define a functor $F : \mathbf{Set}\times\mathbf{Set} \to \mathbf{ASet}$ as follows: $F_0 : (Q,X) \mapsto (Q\times X^*, X, \mu_{Q,X})$. A $\mathbf{Set}\times\mathbf{Set}$ morphism is a pair of maps $(j,\tau) : (Q,X) \to (R,Y)$, i.e., $j : Q \to R$ and $\tau : X \to Y$. The result of applying $F_1 : (Q\times X^*, X, \mu_{Q\times X^*,X}) \to (R\times Y^*, Y, \mu_{R\times Y^*,Y})$ to the morphism (j,τ) is $(j\times\tau^*, \tau)$. Define $\eta : 1_{\mathbf{ASet}} \to U\circ F$ on each object (Q,X) to be $\eta_{Q,X} : (q,x) \mapsto ((q,\epsilon),x)$. Then F is the left adjoint of U, and η is the unit of the adjunction. $F(Q,X)$ is called the free ASet on (Q,X).

Proof. It is routine to check that $F_1(j,\tau)$ is an $\mathbf{ASet}$ morphism. To prove that F is the left adjoint, we have to show for any ASet (R,Y,γ), so $\gamma : R\times Y \to R$, and any pair of maps $(g,\rho) : (Q,X) \to (R,Y)$, where $g : Q \to R$ and $\rho : X \to Y$, that there exists a unique morphism of ASets $\psi : (Q\times X^*, X, \mu_{Q,X}) \to$

(R, Y, γ), such that $(g, \rho) = U(\psi) \circ \eta_{Q,X}$. Such a morphism ψ must consist of a pair of maps, $\psi = (h, \chi)$, where $h : Q \times X^* \to R$, and $\chi : X \to Y$. So, we are looking for a unique morphism $\psi = (h, \chi)$, such that $(g, \rho) = (h, \chi) \circ \eta_{Q,X}$, that is, the following diagram commutes:

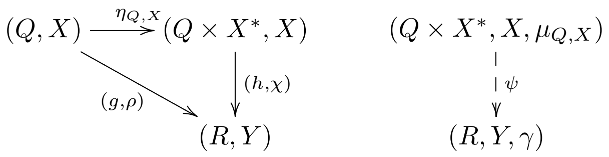

(6)

and since $\psi = (h, \chi)$ is a morphism, such that the following diagram also commutes:

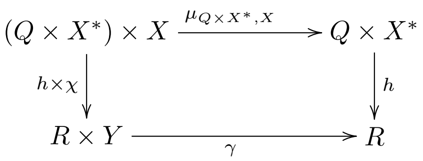

(7)

We have to show that the commutativity of Diagrams 6 and 7 determines ψ uniquely. Diagram 6 says that for all $q \in Q$, $x \in X$

$$(h, \chi) \circ \eta_{Q,X}(q, x) = (g, \rho)(q, x)$$

$$\text{i.e.,} \quad (h, \chi)((q, \epsilon), x) = (g(q), \rho(x))$$

$$\text{i.e.,} \quad (h(q, \epsilon), \chi(x)) = (g(q), \rho(x)).$$

Thus, Diagram 6 forces, for all $x \in X$, $\chi(x) = \rho(x)$, i.e., $\chi = \rho$, and for all $q \in Q$,

$$h(q, \epsilon) = g(q). \tag{8}$$

Equation 8 forms the base part for the recursive definition of h.

Following the clockwise path in Diagram 7 applied to $q \in Q$, $w \in X^*$, $x \in X$ gives us $h \circ \mu_{Q \times X^*,X}((q, w), x) = h(q, w \cdot [x])$, by definition of μ. Following the anticlockwise path in Diagram 7 gives us, since $\chi = \rho$, $\gamma \circ (h \times \chi)((q, w), x) = \gamma \circ (h, \rho)((q, w), x) = \gamma(h(q, w), \rho(x))$. Commutativity of Diagram 7 requires these two paths to be equal, i.e.,

$$h(q, w \cdot [x]) = \gamma(h(q, w), \rho(x)). \tag{9}$$

This equation provides the recursive part of the definition of h. So, if h exists, then it satisfies Equations 3 and 9.

The length of a string w was defined in Diagram 11. It is now straightforward to prove by induction on the length of w that $h(q,w) = \gamma^*(g(q), \rho^*(w))$, for all $q \in Q$, $w \in X^*$, where $\gamma^* : R \times Y^* \to R$ is the run map of (R, Y, γ).

Base: If $\mathit{length}(w') = 0$, $w' = \epsilon$, so $h(q, w') = h(q, \epsilon) = g(q)$. But, $\gamma^*(g(q), \rho^*(w')) = \gamma^*(g(q), \rho^*(\epsilon)) = \gamma^*(g(q), \epsilon) = g(q)$, by definition of the run map γ^*, so in this case $h(g(q), \epsilon) = g(q)$. So, the base case is proven.

Inductive step: If $\mathit{length}(w') > 0$, then $w' = w \cdot [x]$ for $w \in X^*$, $x \in X$, so

$$\begin{aligned}
h(q, w') &= h(q, w \cdot [x]) \\
&= \gamma(h(q, w), \rho(x)) && \text{(Equation 9)} \\
&= \gamma(\gamma^*(g(q), \rho^*(w)), \rho(x)) && \text{(induction hypothesis)} \\
&= \gamma((g(q), \rho^*(w) \cdot \rho(x)) && \text{(definition of } \gamma^*\text{)} \\
&= \gamma((g(q), \rho^*(w \cdot [x]) && \text{(definition of } \rho^*\text{)} \\
&= \gamma((g(q), \rho^*(w')). && \text{(as required)}
\end{aligned}$$

As the base and inductive cases are proven, the principle of induction establishes the result. □

References

1. Arbib MA, Manes EG (1975) Arrows, structures, and functors: The categorical imperative. London, UK: Academic Press.
